# Selective electrochemical reduction of CO_2_ on compositionally variant bimetallic Cu–Zn electrocatalysts derived from scrap brass alloys

**DOI:** 10.1038/s41598-022-17317-6

**Published:** 2022-08-05

**Authors:** Ibrahim M. Badawy, Ahmed Mohsen Ismail, Ghada E. Khedr, Manar M. Taha, Nageh K. Allam

**Affiliations:** 1 Energy Materials Laboratory (EML), School of Sciences and Engineering, The American University in Cairo, New Cairo, 11835 Egypt; 2 Department of Analysis and Evaluation, Egyptian Petroleum Research Institute, Cairo, 11727 Egypt

**Keywords:** Materials science, Nanoscience and technology, Nanoscale materials, Nanoparticles, Energy science and technology, Renewable energy

## Abstract

The electrocatalytic reduction of carbon dioxide (CO_2_RR) into value-added fuels is a promising initiative to overcome the adverse effects of CO_2_ on climate change. Most electrocatalysts studied, however, overlook the harmful mining practices used to extract these catalysts in pursuit of achieving high-performance. Repurposing scrap metals to use as alternative electrocatalysts would thus hold high privilege even at the compromise of high performance. In this work, we demonstrated the repurposing of scrap brass alloys with different Zn content for the conversion of CO_2_ into carbon monoxide and formate. The scrap alloys were activated towards CO_2_RR via simple annealing in air and made more selective towards CO production through galvanic replacement with Ag. Upon galvanic replacement with Ag, the scrap brass-based electrocatalysts showed enhanced current density for CO production with better selectivity towards the formation of CO. The density functional theory (DFT) calculations were used to elucidate the potential mechanism and selectivity of the scrap brass catalysts towards CO_2_RR. The d-band center in the different brass samples with different Zn content was elucidated.

## Introduction

To mitigate the harmful effects of climate change, the scientific community is pursuing a vision for sustainability that falls under the planetary boundaries. High energy demand has led to the overconsumption of fossil fuels and excess carbon dioxide (CO_2_) emissons^1,2^. The excessive mining of minerals, as well, due to general poor regulation is entangled in a web of environmental and societal harm^3,4^. Hence, considerable research work is directed towards steering away from the mining of metals and combustion of fossil fuels into greener alternatives^5,6^. Developing a solution that will make use of renewable energy sources and building a basis for affordable recycling schemes are the key to a sustainable future^7,8^.

Thus, as a promising step towards closing the carbon cycle, the electrocatalytic CO_2_ reduction reaction (CO_2_RR) has garnered vast research interest. Despite proving to be a complex reaction, many studies have demonstrated strong results using a wide variety of electrocatalysts^9–14^. The underlining goal for these studies is to eventually scale this process whereby it can compete with the existing energy production methods. Making decisions aimed towards minimizing both capital and running costs are therefore essential considerations. With regards to the latter, converting CO_2_ directly to higher hydrocarbons (C_2+_) using copper (Cu), the catalyst of choice, is a challenging task. Namely because Cu is not selective and produces a variety of hydrocarbons with poor faradic efficiencies (FE)^15^. From an industrial standpoint, it is preferable to produce a single product at high FE rather than many at low FE because it would spare separation costs. Another challenge is by converting CO_2_ directly into CH_4_ one faces high overpotentials, which can be a significant source of inefficiency^16^. Techno-economic models suggest an indirect pathway for the production of hydrocarbons via carbon monoxide (CO) can be more practical in light of commercial viability^17–19^. Moreover, well-established industrial processes, such as Fischer–Tropsch, utilize direct CO-based feedstock^20^. Thus, CO as a product of CO_2_RR in many respects is more favorable over hydrocarbons.

On the other side of the equation, cheap and abundant catalyst materials must also be considered to minimize capital costs. The best, consistently performing catalysts for CO production are Pd, Au, and Ag^11,21,22^. However, designing a catalyst from noble metals alone is not cost-effective. Brass, on the other hand, is vastly available, cheap, and possesses no environmental concerns. Many studies have demonstrated promising results with brass as an electrocatalyst for CO_2_RR^12,23–31^. A metal–organic framework (MOF) derived electrocatalyst with Cu and Zn bimetallic centers generated CO with 88% FE. The phthalocyanine molecule facilitated the synergism between CuN_4_ and ZnO_4_ centers to produce CO at a high rate^32^. In another study, oxidized brass nanoparticles supported on carbon nanotubes (CNT) were synthesized using a multistep calcination method. They reported ≈ 50% FE for CO and ≈ 90% FE for syngas^33^. Despite the promising performance of brass-based nanoparticles, their scaling can be challenging and costly due to the requirement of specialized chemicals and precise synthesis techniques. Therefore, nanoparticles are not always the most practical from an industrial standpoint^34,35^. Consequently, nanofoams and structures were produced directly from metal foils^5,26,27^. Stojkovikj et al*.* prepared bronze nanofoam via high potential processing. The structure consisted of dendrites that substantially increased the surface roughness, resulting in an improvement in CO FE from 35–40% to ≈ 85%^36^. However, the production of nanofoam requires extremely high current densities, which is a cost burden, especially at the industrial scale^37,38^. To this end, the direct use of metal scraps without the need for expensive processing seems to be the most cost-effective and environmentally sustainable alternative. Moreover, despite the high reported activity of different Cu–Zn-based electrocatalysts in CO_2_RR, the effect of alloying them with another metal on the CO_2_ reduction performance is still lacking^39^.

Herein, we explore the use of scrap brass alloys with different Zn content CO_2_RR as a basis for the development of a new platform of recycled electrocatalysts. The activity and selectivity of brass catalysts were enhanced using inexpensive methods and through the controlled deposition of Ag, which stabilizes ZnO and introduces more active sites for CO production. In addition, density functional theory (DFT) simulations have been employed to help understand the mechanism by which the catalysts facilitate the formation of CO and its selectivity over other products.

## Material and methods

### Materials

Ammonia 25%, ammonium sulfate (≥ 99.0%), and silver nitrate (≥ 99.0%) were obtained from Sigma Aldrich. Potassium bicarbonate (99%) was obtained from Alfa Aesar. The brass alloys of different compositions were cut into 1.5 × 3.0 cm sheets. All reagents were used without further purification. MilliQ ultrapure water was applied throughout the study.

### Pre-treatment of brass alloys

The samples (1-CuZn with 5% Zn, 2-CuZn with 15% Zn, 3-CuZn with 30% Zn, and 4-CuZn with 50% Zn) were polished with 1200 grit sandpaper until a smooth, mostly homogenous surface was obtained. Next, they were sonicated for 2 min, once in ethanol and then in distilled water. Finally, the samples were annealed in the air for one hour at 500 °C with a rate of 10 °C/min. The bulk composition of the samples was analyzed by energy dispersive x-ray (EDX) measurements, see Fig. S5.

### Galvanic replacement

Ag was deposited via galvanic replacement. A solution of 50 mL of 1.68 M NH_4_OH was prepared, then 7.664 g of (NH_4_)_2_SO_4_ and 0.8494 g of AgNO_3_ were added, and the mixture was continuously stirred. The metal scrap was dipped inside the prepared solution for a timed number of seconds.

### Electrochemical measurements and product analysis

The electrochemical CO_2_ reduction reaction was carried out in a glass gas-tight H-type cell with two compartments separated by a proton exchange membrane (Nafion 117). A Biologic SP300 type potentiostat/galvanostat was used for all electrochemical experiments in a 3-electrode set-up. The counter electrode used was a platinum coil, and the reference electrode was Ag/AgCl (KCl sat.). Unless otherwise stated, the electrolyte used for all measurements was aqueous 0.5 M KHCO_3_ for both the cathode and anode compartments, which was bubbled with 99.999% CO_2_ for 30 min. The catholyte was continuously stirred and had a volume of 40 mL. The measured potentials were converted to the reversible hydrogen electrode (RHE) reference scale using the formula. E_RHE_ = E_Ag/AgCl_ + 0.197 V + 0.059 pH. All currents were normalized to the geometric surface area of the electrodes. The pH of the electrolyte was measured to be 7.5. During the chronoamperometry measurements, the gas phase products were quantified by a gas chromatography (SRI 8610C Multi-gas #5, 6′ Haysep D and 6′ Molecular Sieve 5A) that was directly connected to the cathodic compartment via the online sampling loop. The gas chromatography was equipped with a thermal couple detector (TCD), and a flame ionization detector (FID) with Argon (99.9999%) applied as the carrier gas. The liquid phase products were quantified using High-Performance Liquid Chromatography (HPLC, Eclipse XBD-C18) with a flow rate of 0.6 mL/min and HPLC grade acidified deionized water as the mobile phase.

### Materials characterization

The morphology of the brass samples was characterized by Zeiss SEM Ultra 60 field emission scanning electron microscope (FESEM) with 8 kV applied voltage, and the elemental composition was determined using energy dispersive X-ray analysis (EDX) attached to the SEM equipment. Rigaku SmartLab X-ray diffractometer was used to record the grazing incident x-ray diffraction (GIXRD) spectra in the 2θ range of 5°–80° at step rate of 0.007°.

### DFT calculations

For the DFT calculations, the standard Cambridge Serial Total Energy Package (CASTEP) implemented in Materials Studio version 2017 was used^40^. We began with Cu_3_Zn unit cell, then a (3 × 3) supercell was created to simulate the four different structures. A (111) surface was cleaved, which was chosen based on the experimental results. A vacuum slab of 15 Å was included to avoid interaction with its image. For the Ag-coated 3-CuZn, a cluster of four Ag atoms was added on the top layer. The top and bottom layers and the adsorbate were allowed to relax, while the middle two layers were fixed. All spin polarized calculations were performed using the generalized gradient approximation of Perdew, Burke and Ernzerhof (GGA-PBE) with ultrasoft pseudopotential. The cutoff energy was set to 400 eV with a convergence criterion of 5 × 10^–6^ eV/Å, maximum force 0.01 eV/A, maximum stress 0.02 GPa, maximum displacement 5 × 10^–4^ Å and the Monkhorst-pack k-points sampling was set to a 2 × 2 × 1 grid. We also did transition state (TS) search using complete Linear Synchronous Transit/Quadratic Synchronous Transit (LST/QST) protocol with root mean squared (RMS) convergence of 0.01 eV/Å to identify the energy barriers for the reaction. The nudged elastic band (NEB) calculations were done to confirm the transition states of the reaction.

Regarding the effect of the solvent and solvation of CO_2_, the following correction factors were used; − 0.51 eV, 0.13 eV, 0.25 eV and 0.5 eV for the species CO*, CO_2_*, COOH* and OH*, respectively. This is a common practice for CO_2_RR computational modeling as it spares computational cost considerably^41,42^. To further confirm the accuracy of this correction of the PBE-gas phase, we performed pilot calculation for CO* using COSMO solvent model for aqueous solvent with a dielectric constant of 78.54, where the difference was found to be within 0.1.

## Results and discussion

### Characterization analysis

**Figure 1:**
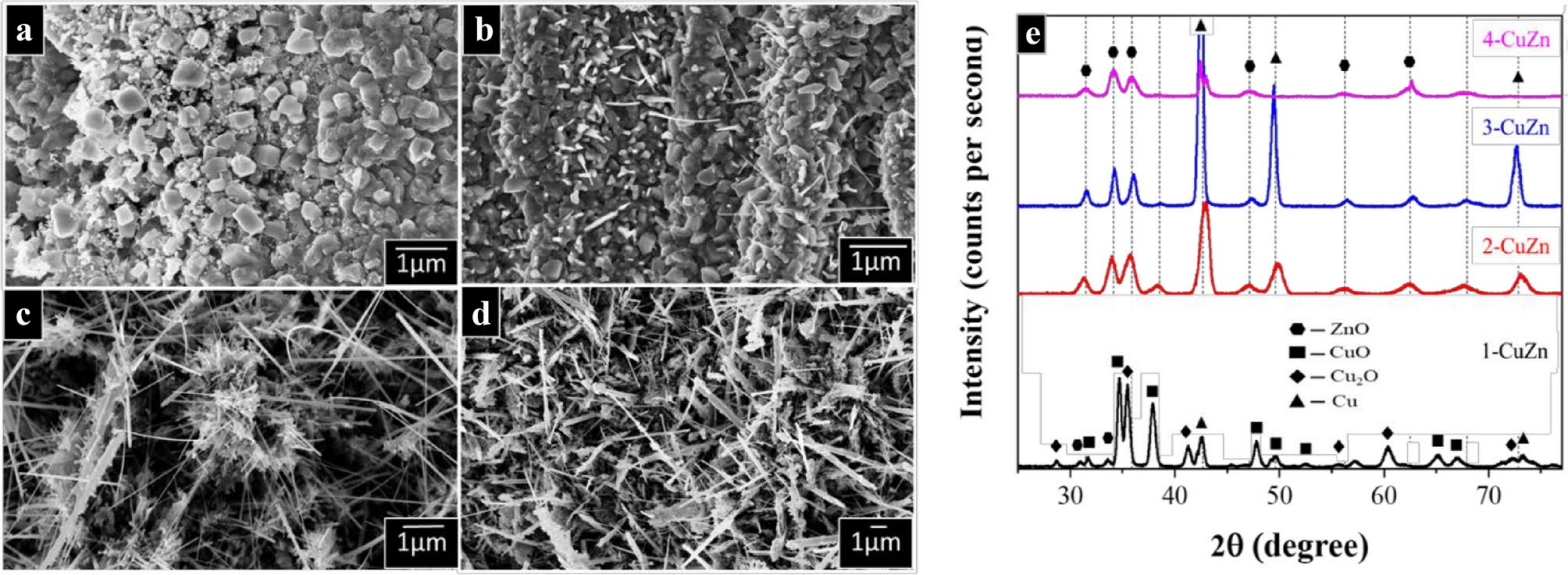
SEM images of the (**a**) 1-CuZn with 5% Zn, (**b**) 2-CuZn with 15% Zn, (**c**) 3-CuZn with 30% Zn, and (**d**) 4-CuZn with 50% Zn samples after annealing in air at 500 °C for 1 h. (**e**) Grazing angle XRD patterns for all 4 samples after annealing at 500 °C for 1 h with 10 °C/min heating rate.

The scrap brass specimens were annealed at 500 °C in the air to oxidize the surface and allow for nanostructuring^43–46^. Upon increasing the Zn content in the brass sample, the annealing resulted in the formation of different nanostructures, see Fig. 1. The sample with the least Zn content (5% Zn) shows the formation of distorted nanocubes on the surface (Fig. 1a), which are typically observed for CuO and Cu_2_O^47^. Upon increasing the Zn content, nanoneedles start to form on the surface of all samples, which may be attributed to the formation of ZnO with hexagonal lattice structure^48,49^. Note that Zn has a higher oxidation affinity than Cu^50^. Therefore, upon heating, Zn atoms diffuse to the surface along the grain boundaries, where they are then oxidized and form the nanoneedle structures. The density of nanoneedles on the surface increases with increasing the Zn content (Fig. 1b–d).

To better understand the crystal structure and composition of the formed phases, glancing angle XRD patterns were recorded. Figure 1e indicates the formation of Cu_2_O and CuO for sample 1-CuZn and to a lesser degree for sample 2-CuZn. The diffraction peaks of Cu_2_O (JCDPS: 96-100-0064) were observed at 29.6° (110), 36.5° (111), 42.3° (200), 62.4° (220), and 73.6° (311). The most pertinent peaks of CuO (JCDPS: 96-110-0029) appeared at 32.66° (110), 35.4° (002), 38.86° (111), 48.9° (− 202), 66.6° (− 311), and 68.4° (220). These peaks were absent for samples 3-CuZn and 4-CuZn. The peaks observed at 31.8° (100), 34.4° (002), 36.2° (101), 56.5° (110), and 62.8° (103) can be assigned to the hexagonal phase of ZnO crystals (JCDPS: 96-153-7876). Although no peaks of metallic Zn were observed, the peak at 44.7° is characteristic of metallic Cu (111), which can be related to the fact that Zn has a higher oxidation potential and is thus more readily oxidized than Cu. As the Zn content in the base alloy increases, it oxidizes quickly, dominating the surface and leaving Cu metal underneath. The estimated grain size from the peak broadening of the ZnO (Table S1) indicates that there is no pronounced difference between the samples. Furthermore, the lattice microstrain, estimated from the peak broadening, is very low, suggesting the little formation of defects in the crystal structure^51,52^.

### Electrochemical CO_2_RR

**Figure 2:**
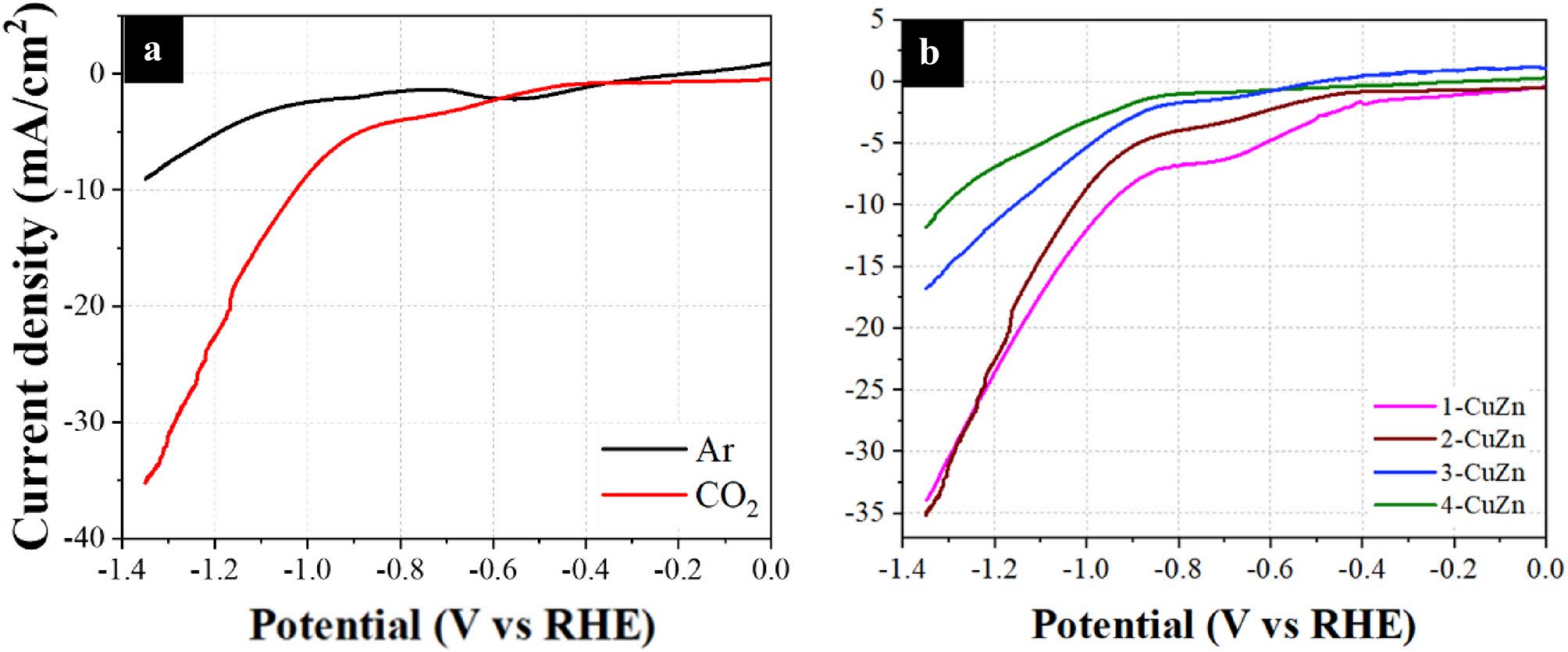
Linear scan voltammetry under continuous CO_2_ and Ar purge in 0.5 M KHCO_3_ and 0.5 M Na_2_SO_4_, respectively for samples (**a**) 1-CuZn, (**b**) 2-CuZn, (**c**) 3-CuZn, and (**d**) 4-CuZn.

The annealed scrap brass samples were implemented directly into the electrochemical system to study their efficiency as catalysts for CO_2_RR. The performance of the brass scraps was first studied using linear sweep voltammetry (LSV), as shown in Fig. 2. The experiments were conducted in CO_2_-saturated 0.5 M KHCO_3_ and in Ar-saturated 0.5 M Na_2_SO_4_ electrolytes under continuous bubbling of the respective gas. The samples were pre-scanned at 10 mV/s for 5 cycles to ensure they reached equilibrium and stabilized before recording their actual response. Figure S1 reveals a broad peak for all the four samples under Ar bubbling in the potential range of − 0.6 to − 0.4 V vs. RHE, which can be attributed to the reduction of Cu_2_O^53^. The curve of sample 4-CuZn, however, shows a different peak between − 1 and − 0.8 V vs. RHE, which is likely ascribed to the reduction of ZnO^53^. Table S2 shows the extracted data from the LSV curves for each sample. It is also worth noting that at 0 V vs. RHE, the electrocatalysts showed non-zero current, likely because of double-layer charging and parasitic reactions at the surface of the electrode. It is difficult to eliminate these effects as LSV cannot distinguish between electrocatalyst, electrolyte, and charge transfer events^54^.

**Figure 3:**
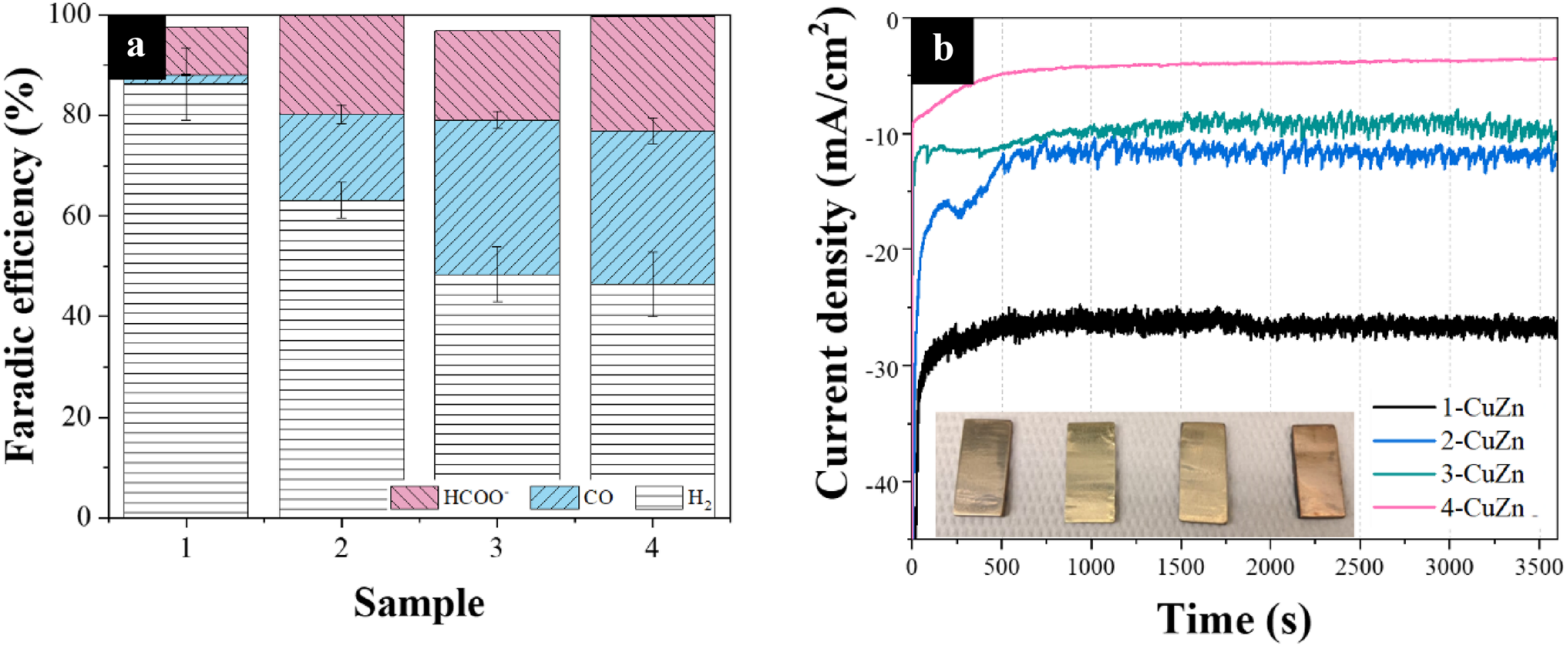
The electrocatalytic performance of the samples at a potential of − 0.91 V vs. RHE. (**a**) The measured Faradic efficiencies and (**b**) the chronoamperometry profiles with an inset of a photograph of the 4 polished brass samples.

The onset potential for the samples tested under CO_2_ shifts towards more negative potentials as the Zn content increases (from − 0.17 to − 0.851 V vs. RHE). This is likely due to the ability of Zn to suppress the hydrogen evolution reaction (HER), which is observed in Fig. 3a as well, where the FE of H_2_ is much less for the samples with high Zn content as compared to the samples with lower Zn content^55,56^. Not to mention that the difference in the current density generated between the curves under CO_2_ bubbling and Ar bubbling progressively becomes more pronounced as the Zn content increases. This is strong indication that most of the current density generated by sample 1-CuZn in the LSV is due to HER where the difference between the CO_2_ condition and Ar is least. Nevertheless, the onset potential is only a guiding parameter that should not be over-interpreted because it can be influenced by parasitic reactions and system sensitivity^57^. All in all, the addition of Zn to Cu, despite increasing the onset potential, minimizes the production of HER.

The surface roughness (Table S3) coincides quite well with the obtained current density for each sample. Sample 1-CuZn has the highest roughness of 134.5 and, as a result, demonstrates one of the highest total current densities of 30 mA/cm^2^ at − 0.91 V vs. RHE. However, most of this current is consumed by the HER, which is a high competitor with CO_2_RR (Supplementary information Fig. S3). It is worth mentioning that surface roughness often influences the performance of the catalyst beyond just the value of the current density, namely the product selectivity. Hence, the early development of current observed in the LSV is most likely attributed to HER overwhelming the surface rather than CO_2_RR^15,58,59^. In our case, changing the Zn content is not only influencing the chemical nature of the catalyst but also the physical nature, i.e., the electrochemical active surface area (EASA)^60,61^. CO_2_RR relies on complicated mass transport equilibria within the buffer electrolyte and, as such, faces many kinetic barriers^62,63^.

Figure S3 shows the calculated FE of each of the samples over the measured potential range, which reveals a weak dependency between the FE and applied potential. Figure 3a shows the FEs at − 0.91 V vs. RHE. Samples 3-CuZn and 4-CuZn show the highest CO FE, reaching 30.8% and 30.6%, respectively, which means both samples share similar product selectivity. The difference of product selectivity between samples 3-CuZn and 4-CuZn is insignificant and the progression of Zn content with respect product FE leads to a plateau rather than a volcano shape. This suggests a saturation of activity with the addition of Zn beyond 30%. As for the reaction rate, sample 3-CuZn has the larger total current density yielding an average of 10.16 mA/cm^2^ at − 0.91 V vs. RHE throughout the 3600 s compared to 3.66 mA/cm^2^ for the 4-CuZn sample as shown in Fig. 3b. The decreased rate activity of 4-CuZn compared to 3-CuZn has been further investigated using density functional theory (DFT) simulations as discussed in a subsequent section.

**Figure 4:**
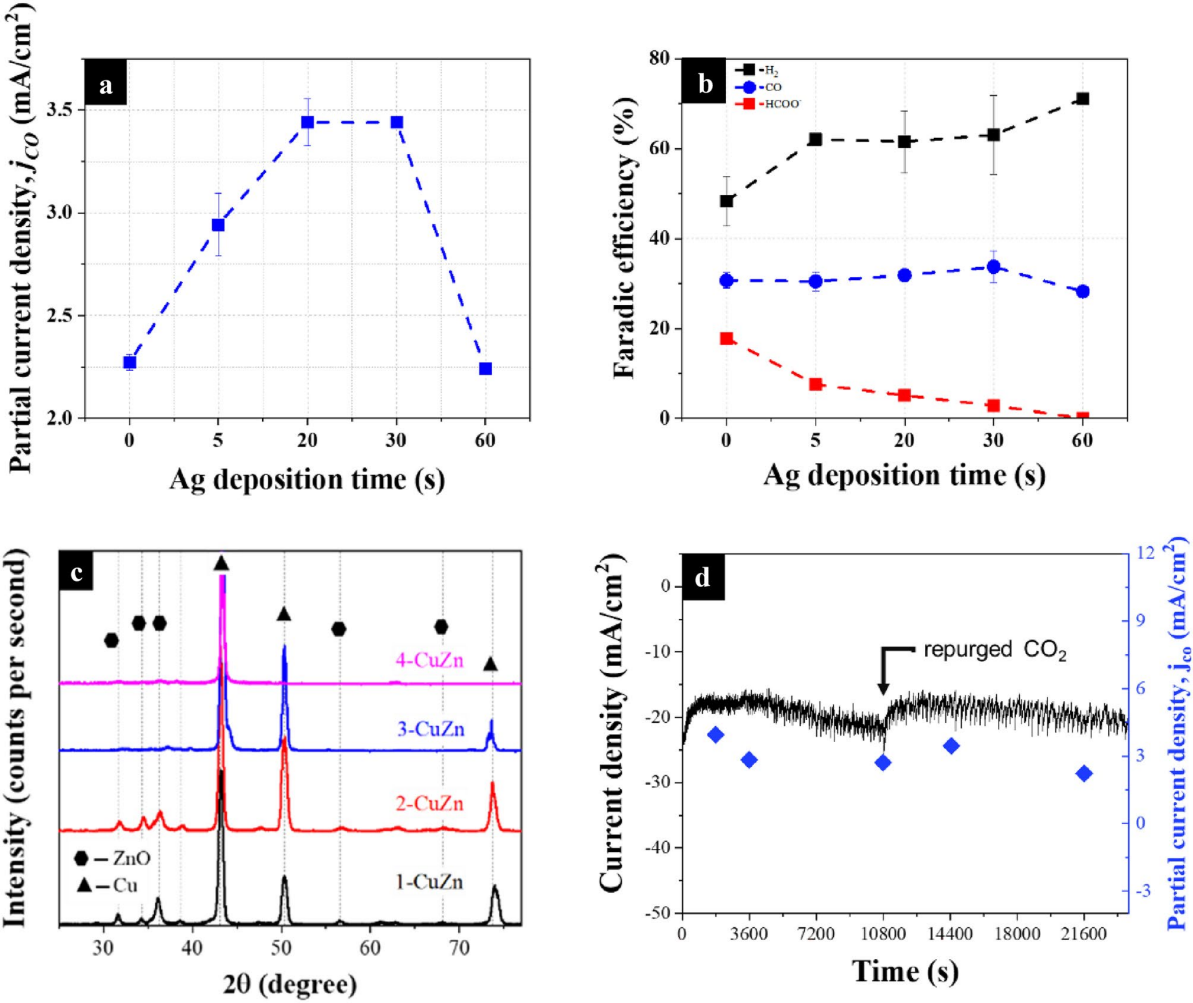
(**a**) The partial current densities of CO across different Ag deposition times at − 0.91 V vs. RHE for sample 3-CuZn (**b**) Faradic efficiency and the ratio of product gases of 3-CuZn with different Ag depositions times at − 0.91 V vs. RHE (**c**) Grazing angle XRD patterns for all samples after electrochemical CO_2_RR at − 0.91 V vs. RHE (**d**) stability showing the current density and partial current density for 3-CuZn with 20 s Ag deposition at − 0.91 V vs. RHE.

It is well-established in the literature that Zn-based electrocatalysts tend to produce HCOO^−^ as a liquid product, which is also observed with our brass electrocatalysts upon HPLC analysis of the electrolyte^26,64,65^. The results in Fig. 3a show that the sample with the least Zn content (1-CuZn) produced the least FE for HCOO^−^ at 9.68%, while the sample with the highest Zn content (4-CuZn) produced the highest FE at 22.6%. Note that CO production likely takes place on the ZnO sites, while HCOO^−^ production takes place on the reduced ZnO sites^66,67^. As a − 1.10 V vs. RHE potential (i.e., the reduction potential of Zn(II) at this particular pH) is applied, ZnO would always be reduced during the CO_2_RR experiment. In our case, CO_2_RR begins at − 1.16 V vs. RHE, which makes the production of HCOO^−^ nearly unavoidable. This is further revealed from the XRD patterns in Fig. 4c of the samples after electrolysis, revealing the almost complete disappearance of the ZnO peaks, especially for samples 3-CuZn and 4-CuZn. This makes the selectivity control with respect to Zn very difficult and urges more designs to overcome such a challenge. The Cu_2_O and CuO have also disappeared due to the applied reduction bias. The state of the Cu however is likely a partially reduced phase between Cu_2_O and Cu metal as previously reported in the literature^54^.

### Modification by Ag

**Figure 5:**
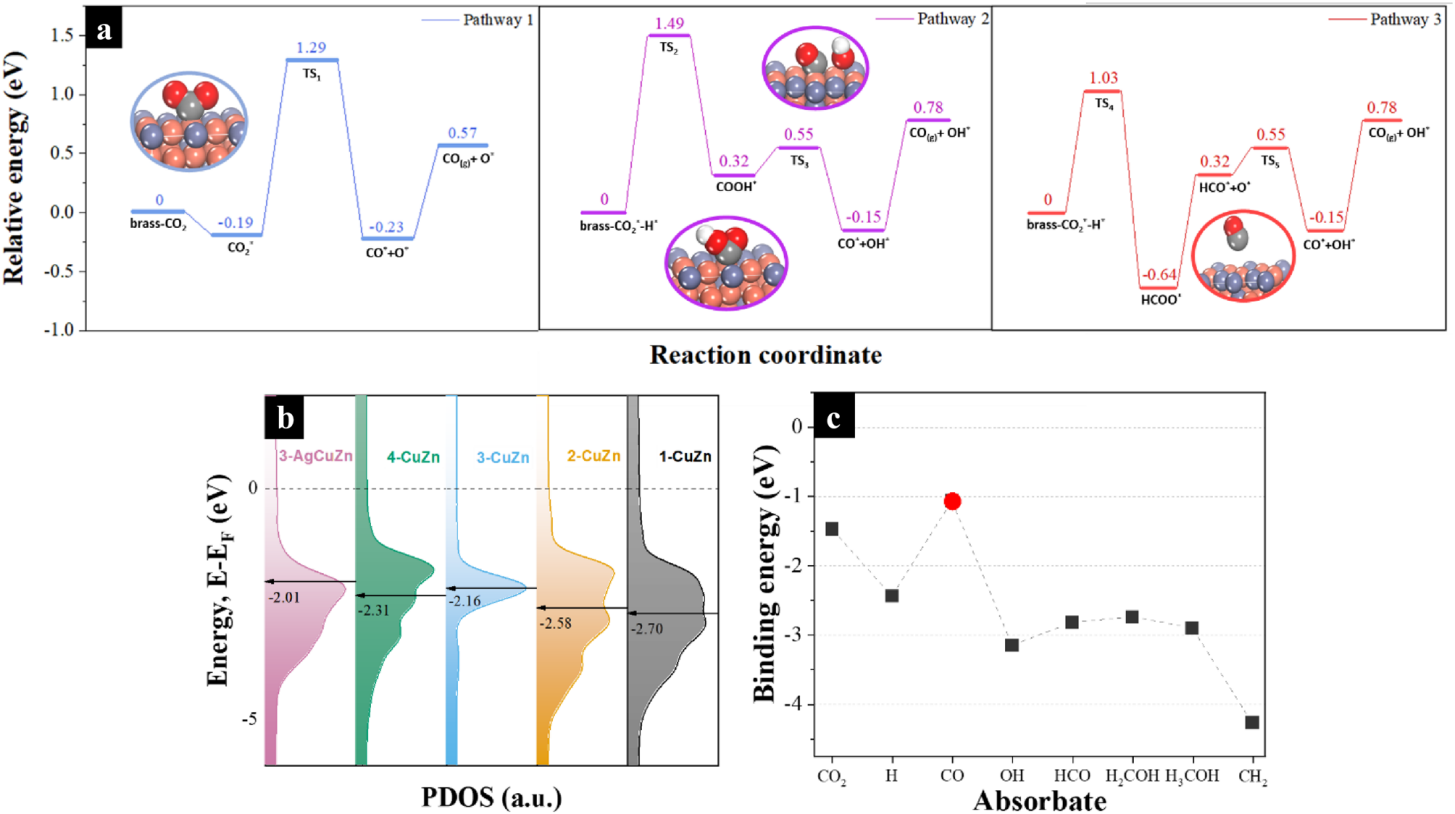
(**a**) Potential energy profile of the mechanism for the three proposed pathways for CO formation on sample 3-CuZn: direct dissociation, H-assisted via COOH*, and via HCOO*. (**b**) The projected density of states (PDOS) of the Cu atom in the four samples on the (111) plane and the d-band center represented by the black arrow while the Fermi level is the dotted line, and (**c**) the binding energy of the different adsorbates during CO_2_RR on the Ag–CuZn (111) surface, the red dot is the binding energy of CO.

To improve the selectivity, Ag was deposited on the surface of the brass samples with the assumption that Ag would stabilize the oxide species where its reduction would be minimized as well as introduce more active sites for CO production. Upon Ag deposition, sample 3-CuZn showed the best FE for CO production. The successful deposition of Ag was confirmed by the new Ag diffraction peaks that appeared at 39.56° (111), 45.74° (200), and 66.11° (220), as depicted in Fig. 5c. A new diffraction peak emerged at 44.34°, which is characteristic of AgZn, revealing the strong affinity of Ag towards Zn that is expected to impact the chemical nature of the overall electrocatalyst during the CO_2_RR^68^. Indeed, the results depicted in Fig. 4b, show the decrease in the formation of HCOO^−^ from 21.6 to 7.53% after 5 s, and to 4.68% after 20 s until it becomes undetectable by the HPLC after 60 s. The XRD pattern (10) in Fig. 2c also shows the preservation of the ZnO peaks after CO_2_RR. Furthermore, Fig. S4a,b shows the SEM images of sample 3-CuZn after Ag deposition as indicated by the emerged rhombic features. Note that those features are retained after the CO_2_RR, revealing the role played by Ag in stabilizing ZnO, whereby it is able to withstand the reduction potentials of CO_2_RR. Ag can also promote CO production by introducing more active sites, as revealed in Fig. 4a. As the Ag deposition time increases, the partial current density, which can be approximated to the rate of CO production, also increases until reaching a maximum of 3.44 mA/cm^2^ in the time interval of 20–30 s. Thus, the addition of Ag improved the selectivity of 3-CuZn towards CO production. To complete the picture for the Ag deposited on brass, a stability measurement was performed as shown in Fig. 4d. The measurement was performed for 6 h, where the electrocatalyst maintained good stability throughout the test.

### DFT modeling and analysis

As previously discussed, annealing the scrap brass samples in the air tends to form electrocatalysts that favor the formation of CO and CHOO^−^. In this regard, DFT calculations were performed to gain insights into the mechanism of the reaction as well as to explain the difference in activities between the samples^69^. CuZn (111) surface was studied in particular because it consistently showed the strongest peaks in the XRD spectra of the annealed samples (Fig. 1c). The sequence of CO_2_RR on the brass surface proceeds as depicted in Fig. 5a. Upon CO_2_ adsorption, Mulliken's charge analysis reveals the charge transfer from 3-CuZn (111) surface to the CO_2_ molecule. As a result, the CO_2_ molecule gains a negative charge of 0.52 e and forms a negatively charged CO_2_ anion. This leads to strong adsorption on the catalyst surface. As the reaction continues, the two C–O bond lengths are elongated, and the (O–C–O) angle is decreased from 180° to 135.9°, revealing the activation of CO_2_. To unveil the mechanism of the dissociation of CO_2_ on the 3-CuZn (111) surface, the following three possible routes for dissociation were elucidated: the direct dissociation and the H-assisted dissociation (the hydrogen source is the electrolyte in this case)^70,71^. Figure 5a shows the direct dissociation route, where the interaction between the 3-CuZn (111) surface and CO_2_ spontaneously forms CuZn-CO_2_* that is then dissociated to CuZn-CO* + O* via the transition state (TS_1_). This process is exothermic by − 0.04 eV and has an activation barrier of 1.48 eV. After that, absorption of 0.8 eV from ambient heat leads to the desorption of CO* from the surface to form CO gas. The second possible dissociation route is the H binding. In the presence of hydrogen, CO_2_ may be activated towards the formation of COOH* or HCOO*. In the case of carboxyl formation, hydrogen atom attacks one of the oxygen atoms of the CO_2_ and forms O–H bond, as depicted in Fig. 5a. This step passes through a transition state (TS_2_) of 1.49 eV. This step is endothermic by 0.32 eV followed by breaking of the C–O bond and forming adsorbed CO* and OH* via (TS_3_) of 0.55 eV. This process is exothermic by 0.47 eV. Concerning the formation of formate route, hydrogen atom binds to the carbon atom of CO_2_ through a transition state (TS_4_) of 1.03 eV, which is an exothermic process of 0.64 eV. Then it splits into HCO* and O* followed by hydrogen transfer to the O* to form CO* and OH* through a barrier of 0.55 eV. The adsorbed CO* desorbs in the gaseous form by 0.93 eV. Although both pathways are viable, our results reveal that the direct dissociation is more thermodynamically favorable. It is also important to note that this particular catalyst surface does not promote C–C coupling, which also would explain the lack of C_2+_ products in the experimental tests. The three mechanisms are shown in more detail in supplementary information Figs. S10–S14.

In order to better understand the selectivity and activity of the electrocatalysts, calculations of the electronic states for all the four different samples (1-CuZn, 2-CuZn, 3-CuZn, 4-CuZn) and Ag-coated 3-CuZn were carried out. Figure 5b shows the projected density of states (PDOS) for Cu atom in each system and its d-band center (ε_d_). The position of the d-band center with respect to the Fermi level is important because it indicates the availability of electrons in the atom^72^. Thus, ε_d_ is strongly correlated to the binding energy, and it is often used as a metric for catalytic activity^73^. A ε_d_ that is closer to the Fermi level pushes the antibonding states above the Fermi level where they will be less filled and hence stronger binding between the catalyst and the absorbed intermediates^74,75^. The four samples along with the Ag deposited sample show relatively close ε_d_ values as seen in Fig. 5b. The largest difference in the samples’ d-band is 0.69 eV between 3-AgCuZn and 1-CuZn which suggests that the electronic effect on the performance of the electrocatalysts might not have profound overall impact^42,76^. The more dominate parameter is perhaps the geometric differences between each of the catalysts’ nanostructures as discussed in characterization analysis. This coincides with the experimental findings in a very specific way; the addition of Ag to the best performing sample, 3-CuZn, did not impact the FE towards CO significantly but rather impacted the partial current density for CO. This enhanced activity for CO specifically is likely owed to the structural changes induced by the Ag rather than the chemical.

As for the selectivity (i.e. the chemical nature) towards CO production specifically, Fig. 5c depicts the binding strength of the different adsorbates that are likely to form during the CO_2_RR process on the most energetically favorable site on the 3-AgCuZn surface. The CO molecule, which is represented by the red dot, has the least binding energy among all the other possible products especially the products that are rank higher in electron transfers. This suggest that its desorption of CO after its formation is the more likely event rather than its further reaction. This is indeed what is observed experimentally as the formation of formate is decreased substantially while the formation of CO is enhanced.

## Conclusion

A simple, inexpensive, and scalable method to repurpose scrap brass metal alloys for CO_2_RR to produce syngas at a reasonable rate was demonstrated. Scraped brass samples with different Zn content were activated via annealing in an air atmosphere, where various nanostructures emerged. Increasing the Zn content led to the formation of denser nanoneedles. As revealed via the XRD analysis, the formation of ZnO was found to inhibit the HER and promote the formation of both CO and CHOO. The Faradic efficiency (FE) of the samples showed weak dependency on voltage. This should be beneficial from an industrial perspective as it is hard to control the working electrode potential in real electrolyzers, where no reference electrodes are being used. The 3-CuZn electrocatalyst with 30% Zn was found to be the best performing among all tested samples with FEs of 30.7% and 21.6% for CO and CHOO^−^, respectively. The addition of Ag improved the performance of the electrocatalyst by increasing the partial current density towards the formation of CO. The DFT calculations confirmed the obtained experimental results in terms of possible formed intermediates and calculated d-band center (ε_d_). The brass alloy modified by Ag showed the lowest binding energy for CO indicating its facile release and preferred production over any other product.

## Supplementary Information

Supplementary Information.
