# Supplementary material for: Selective electrochemical reduction of CO2 on compositionally variant bimetallic Cu–Zn electrocatalysts derived from scrap brass alloys
Source: Sci Rep. 2022 Aug 5;12:13456. doi: 10.1038/s41598-022-17317-6 (PMC9355942; doi:10.1038/s41598-022-17317-6)
Supplement: Supplementary file 1 — Supplementary Information. [file 41598_2022_17317_MOESM1_ESM.docx]

**Supporting Information**

**Selective Electrochemical Reduction of CO_2_ on Compositionally Variant Bimetallic Cu-Zn Electrocatalysts Derived from Scrap Brass Alloys**

Ibrahim M. Badawy, Ahmed Mohsen Ismail, Ghada E. Khedr, Nageh K. Allam*

Energy Materials Laboratory (EML), School of Sciences and Engineering, The American University in Cairo, New Cairo 11835, Egypt

* Corresponding Author’s email: nageh.allam@aucegypt.edu

**Electrochemical set-up**

The CO_2_RR was performed in a tradition H-cell. The H-cell was in a closed loop with the GC where a peristaltic pump was used to circulate the gaseous produced into the injection loop of the GC. The headspace and tubing volume was exactly 38mL. Because the system is closed, the amount of CO_2_ gas available for reaction was fixed and could not be replenished after it would deplete. Thus, long duration tests such as stability were difficult to execute as it would not provide representative results for the electrocatalysts’ performance.

**Extrapolated data from GIXRD**

**Table S1.** Grain size and strain of the ZnO on the surface of the samples estimated after Rietveld refinement and extrapolated using Williamson-Hall plot

| Sample | R_wp_ (%) | Rp (%) | Microstrain on ZnO | Grain size of ZnO (Å) |
| --- | --- | --- | --- | --- |
| 1-CuZnO | 10.175 | 6.925 | - | - |
| 2-CuZnO | 16.88 | 9.884 | 0.00425 | 132.8859 |
| 3-CuZnO | 12.002 | 7.753 | -0.00506 | 116.5685 |
| 4-CuZnO | 12.177 | 8.638 | -0.0016 | 130.5085 |

**LSV measurements**

**Table S2.** Potential and current data extrapolated from LSV. The onset was taken to be the highest cathodic potential when the current began to develop for each sample

| Sample | Onset potential-Ar (V vs RHE) | Onset potential-CO_2_ (V vs RHE) | Current density at -0.91V vs RHE-Ar (mA/cm^2^) | Current density at -0.91V vs RHE-CO_2_ (mA/cm^2^) |
| --- | --- | --- | --- | --- |
| 1-CuZnO | -0.170 | -0.170 | -16.6 | -30.35 |
| 2-CuZnO | -0.529 | -0.583 | -7.57 | -31.02 |
| 3-CuZnO | -0.565 | -0.655 | -9.54 | -14.8 |
| 4-CuZnO | -1.118 | -0.851 | -1.65 | -9.71 |


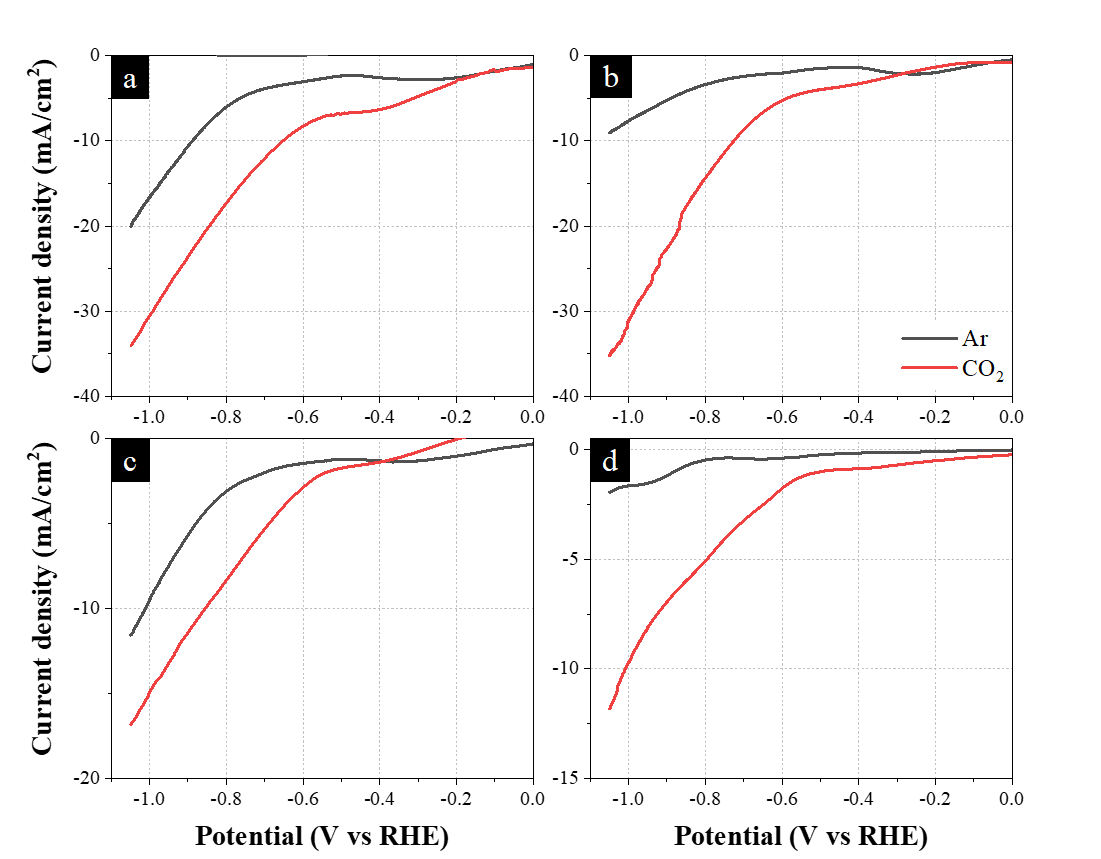


**Figure S1.** Linear scan voltammograms under continuous CO_2_ and Ar purge in 0.5 M KHCO_3_ and 0.5 M Na_2_SO_4_ for samples (a) 1-CuZnO, (b) 2-CuZnO, (c) 3-CuZnO, and (d) 4-CuZnO.

**Electrochemical Active Surface Area (ECSA)**


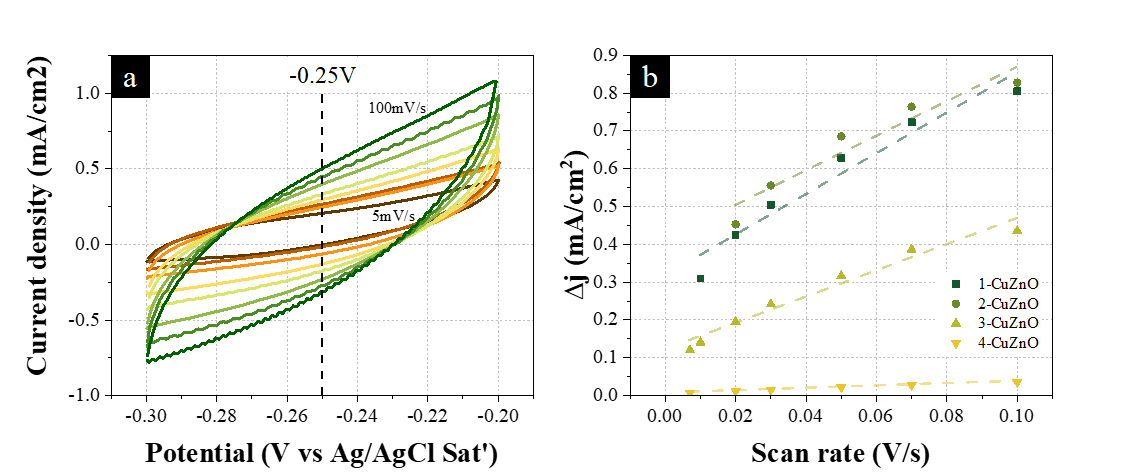


**Figure S2**. Electrochemical active surface area (EASA) calculations derived from (a) the CVs in the non-faradic region for 1-CuZnO between -0.2 and -0.3V *vs* Ag/AgCl and (b) linear regression between the current density difference and scan rate.

The electrochemical active surface area (EASA) was calculated using the following formula:

EASA=C_DL_/C_S_ (1)

where C_DL_ is the capacitance in the non-Faradic region and C_S_ is the specific capacitance taken to be 40 µF/cm^2^ according to Connor *et al.*^1^.

**Table S3.** Extrapolated EASA and roughness from Figure S1

| Sample | C_dl_ (F/cm^2^) | EASA (cm^2^) | Roughness | R^2^ |
| --- | --- | --- | --- | --- |
| 1-CuZnO | 0.00538 | 134.5 | 134.5 | 0.94512 |
| 2-CuZnO | 0.00457 | 114.25 | 114.25 | 0.92105 |
| 3-CuZnO | 0.00347 | 86.75 | 86.75 | 0.96413 |
| 4-CuZnO | 3.13E-04 | 7.8215 | 7.8215 | 0.99563 |

**Faradic efficiency profiles of all samples**


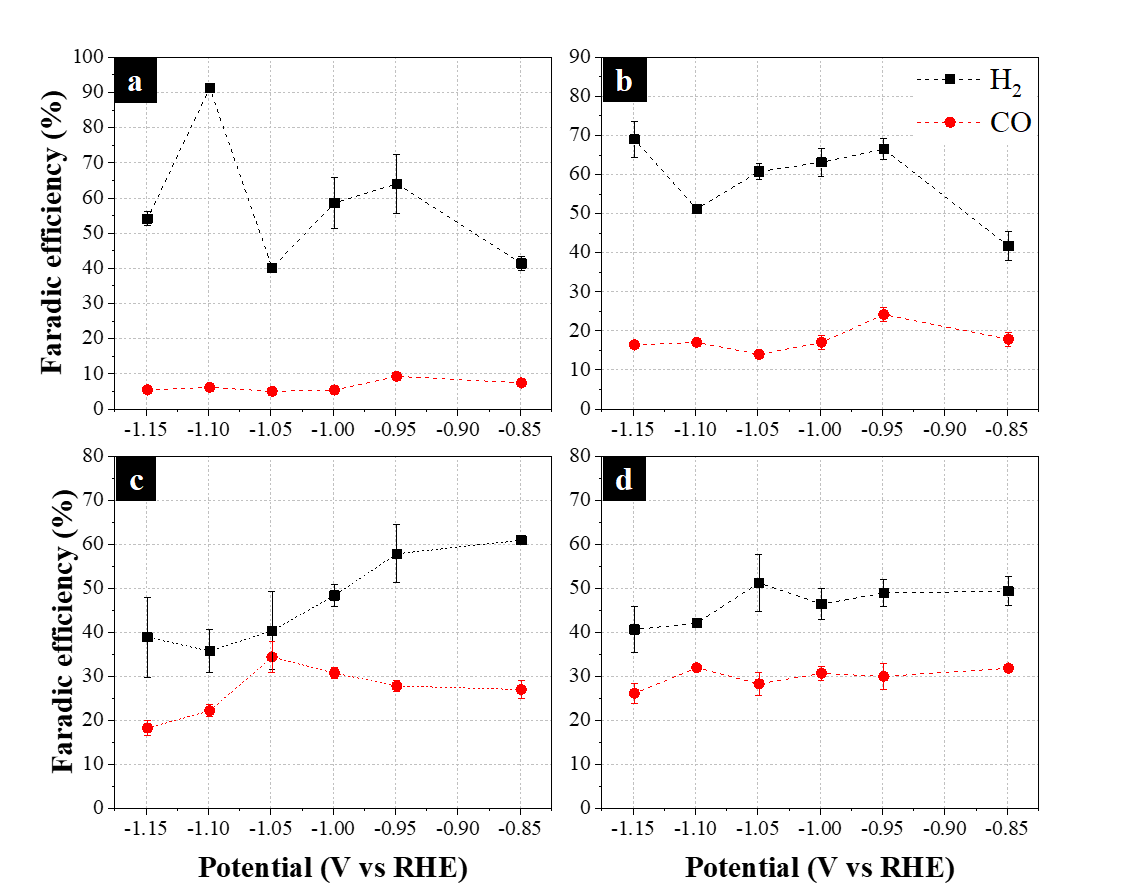


**Figure S3.** Faradic efficiency profiles vs applied potential after 1 h in 0.5M KHCO_3_ for samples (a) 1-CuZnO, (b) 2-CuZnO, (c) 3-CuZnO, and (d) 4-CuZnO.

**Addition of Ag to 3-CuZn**


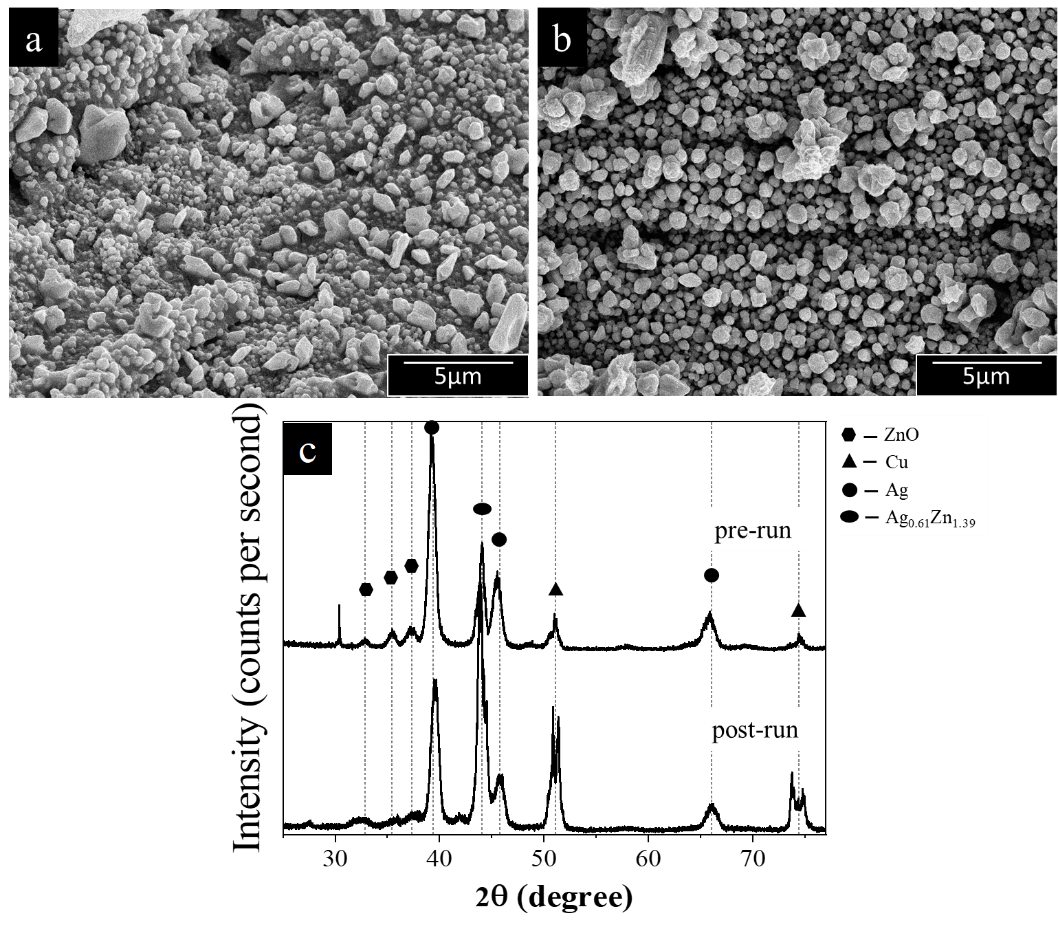


**Figure S4.** SEM images showing the sample 3-CuZnO after 20 s Ag deposition: (a) before electrochemical run and (b) after at -1.00V vs RHE sample (c) 3-CuZn with 20 s Ag deposition before and after electrochemical CO 2 RR at -1.0 V vs RHE.

**EDX spectra**


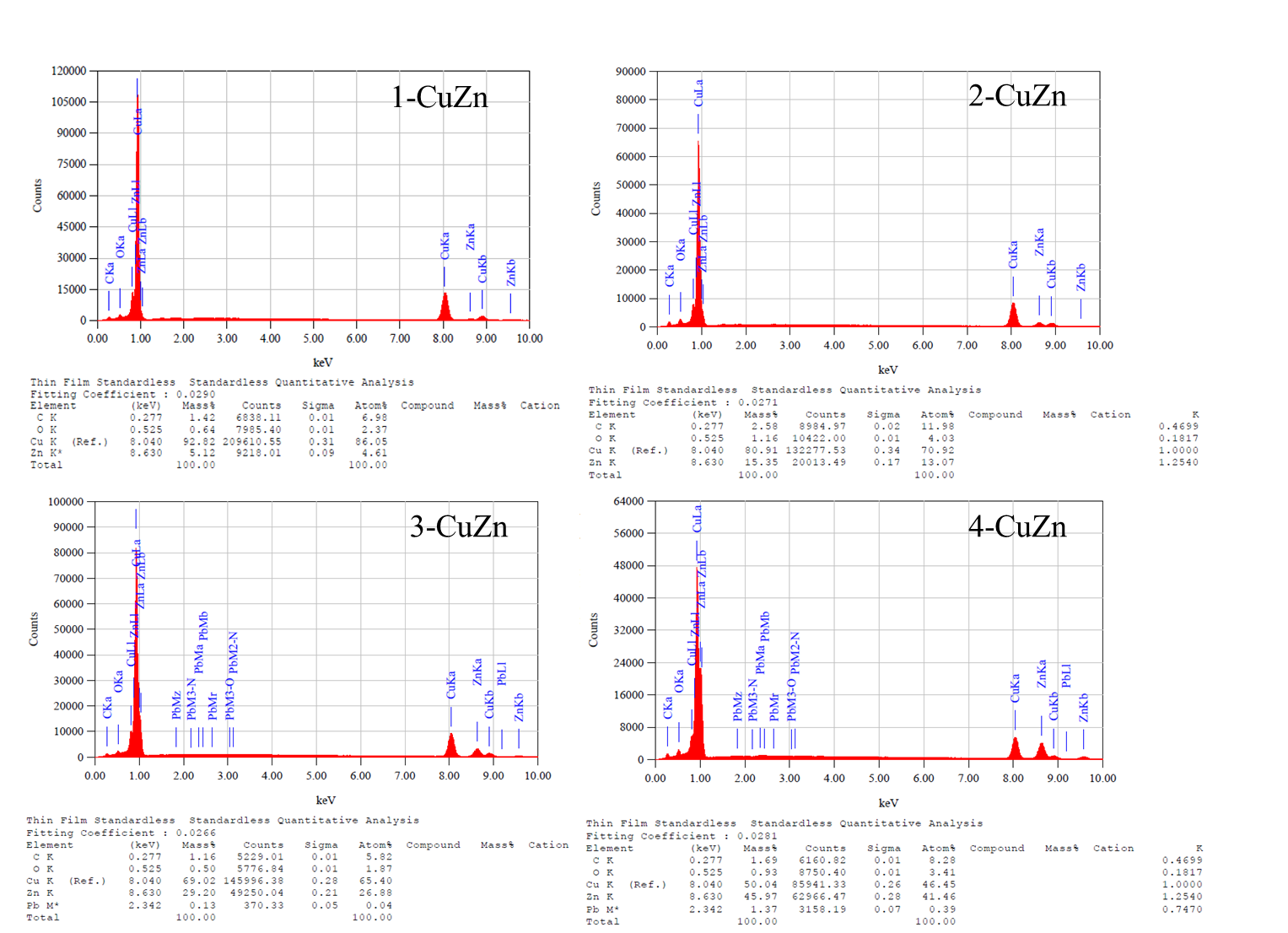


**Figure S5**. EDX measurements of the content of Cu and Zn for the bare, cleaned and polished samples.

**GC, HPLC sample data and Williamson-Hall plot**


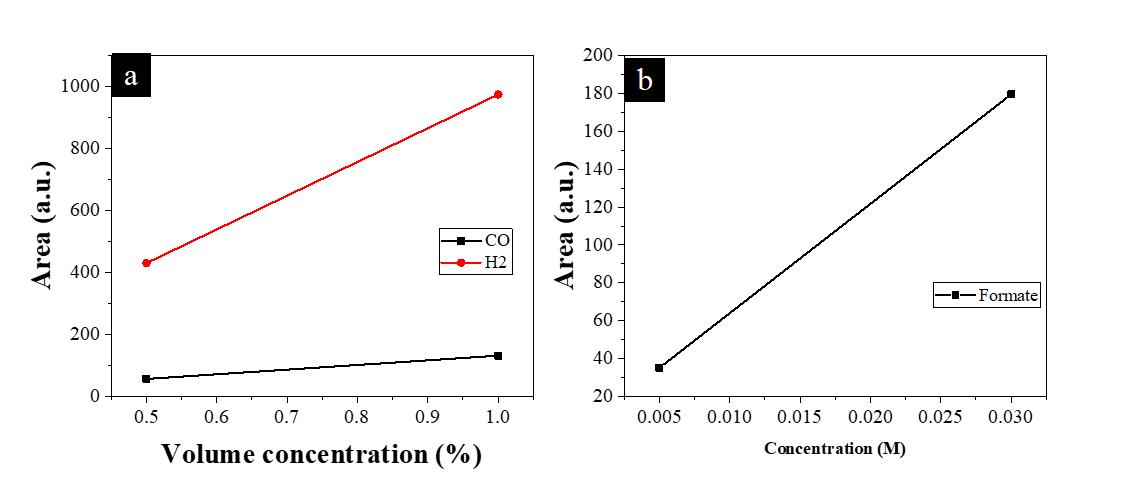


**Figure S6**. a) calibration curves for H_2_ and CO quantification using GC and b) calibration curve for the formate quantification using HPLC


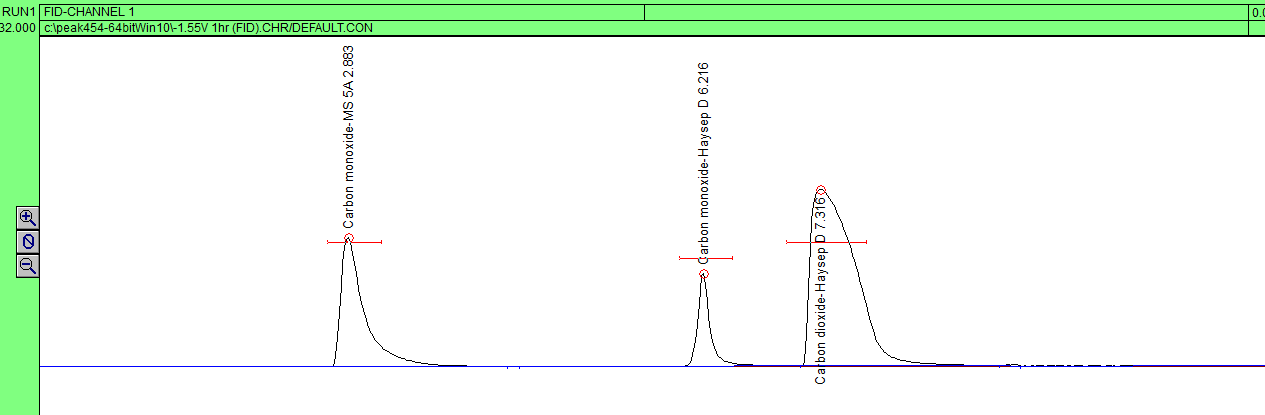


**Figure S7.** Example chromatogram from the GC for the FID reading


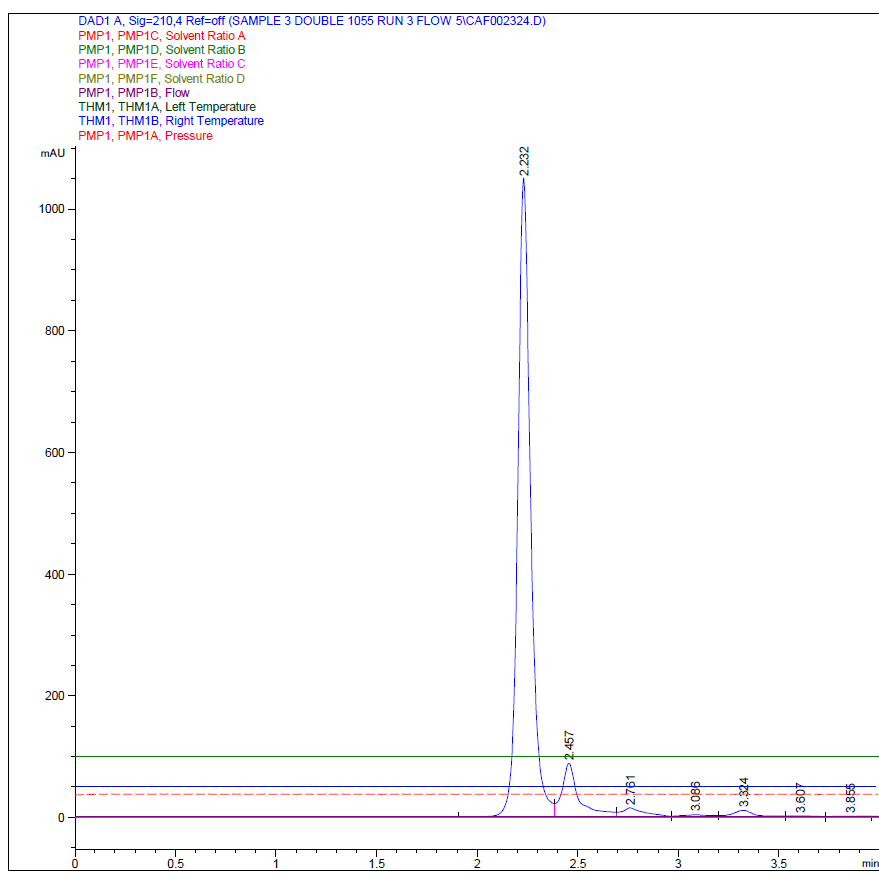


**Figure S8.** Example chromatogram from the HPLC for formate detection. The formate peak is at 2.761mins.

**Figure S9**. Example of the Williamson-Hall plot fitting extrapolated from the diffractogram

**Additional computational details**

The nudged elastic band (NEB) calculations were done via TS confirmation to be sure from the transition states. We started with Cu3Zn unit cell. It is a face centered cubic (FCC)^2^. A (3×3) supercell with a vacuum slab of 15 A was created to simulate the four different concentrations included in our work. The top and bottom layers and the adsorbate were allowed to relax, while the middle two layers were fixed. The free CO_2_ molecule was optimized in a 10×10×10 A˚ unit cell. The optimized C–O bond length and O–C–O angle are 1.178 A˚ and 180.0˚, respectively, which are matched with the available experimental and theoretical data. The adsorption energy of CO2 is calculated as follows:

Eads = ECO_2_ + surface - [ECO_2_ + Esurface]

where E_CO2_ + surface represents the energy of the CuZn(111) surface with the adsorbed CO_2_, E_CO2_ represents the energy of free CO_2_, and E_surface_ represents the energy of the CuZn(111) surface. The Cu site was chosen for adsorption as it is the more favorable one according to the report by Varandili et al.^3^


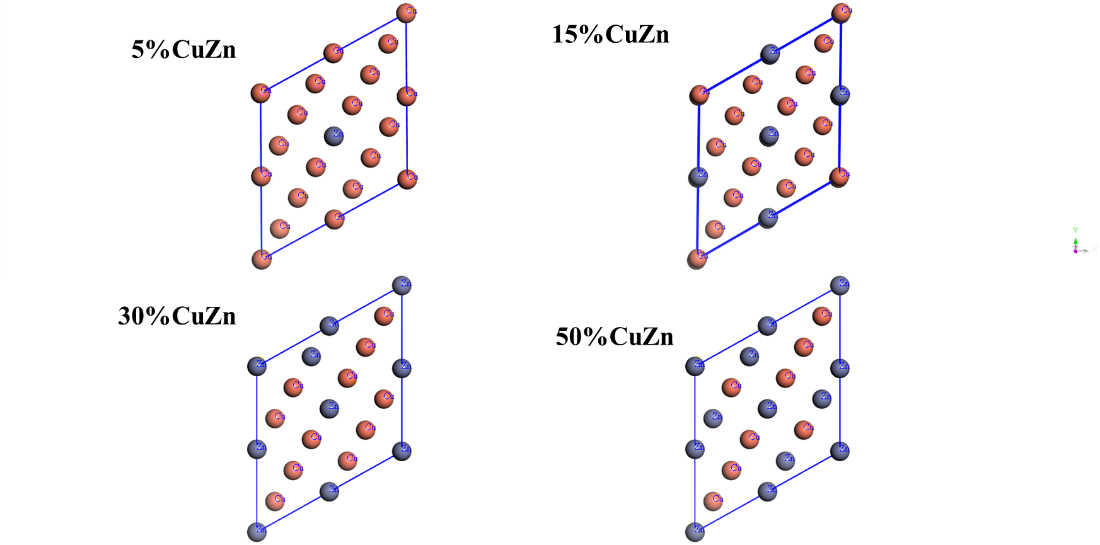


**Figure S10.** Crystal structures for different ratios of the CuZn (111) surfaces.


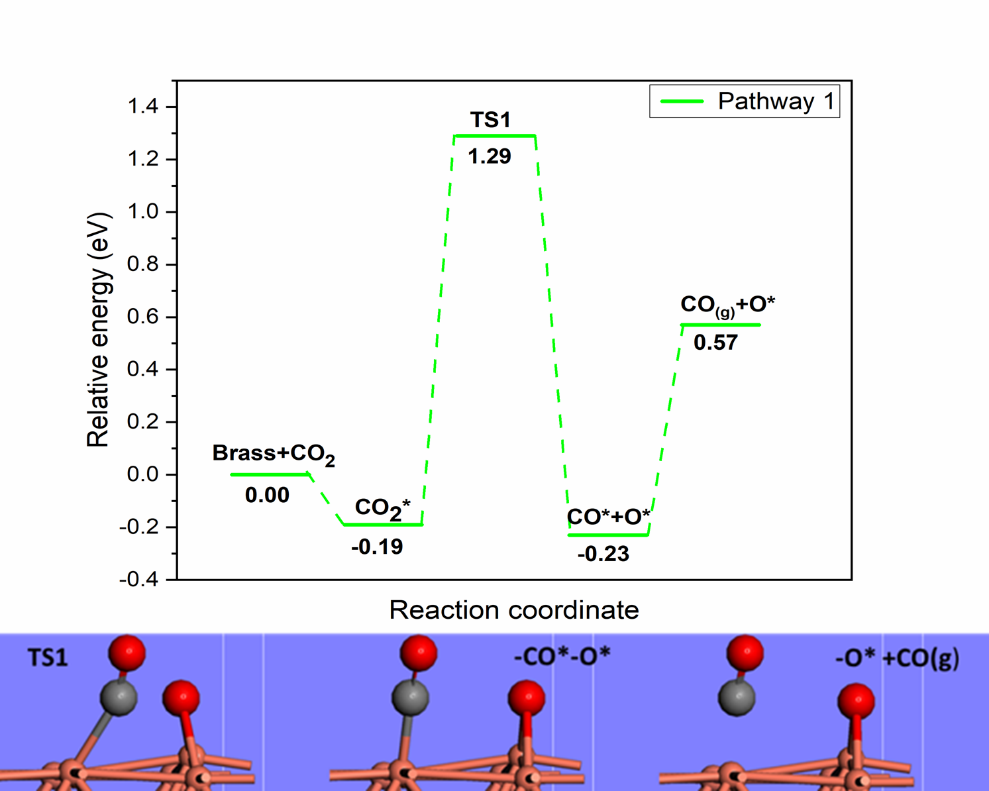


**Figure S11.** Potential energy profile for the **direct dissociation** of CO_2_ on the CuZn30% (111) surface.


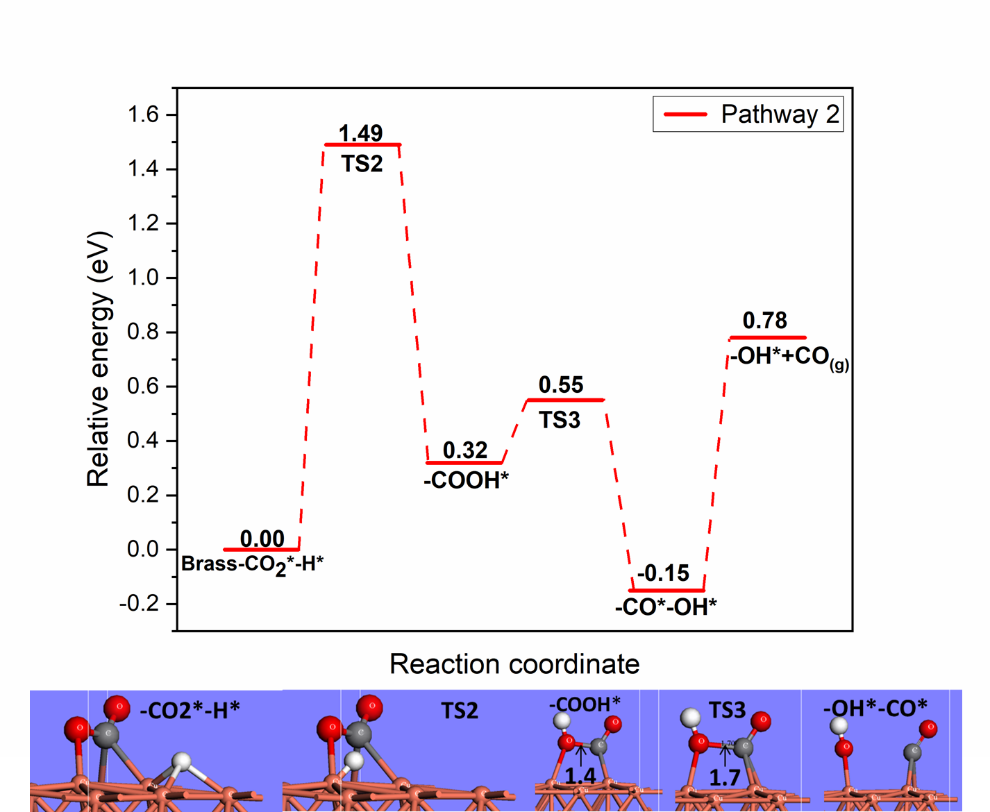


**Figure S12.** Potential energy profile for the **H-assisted dissociation** of CO_2_ via the **COOH*** route on the CuZn30% (111) surface.


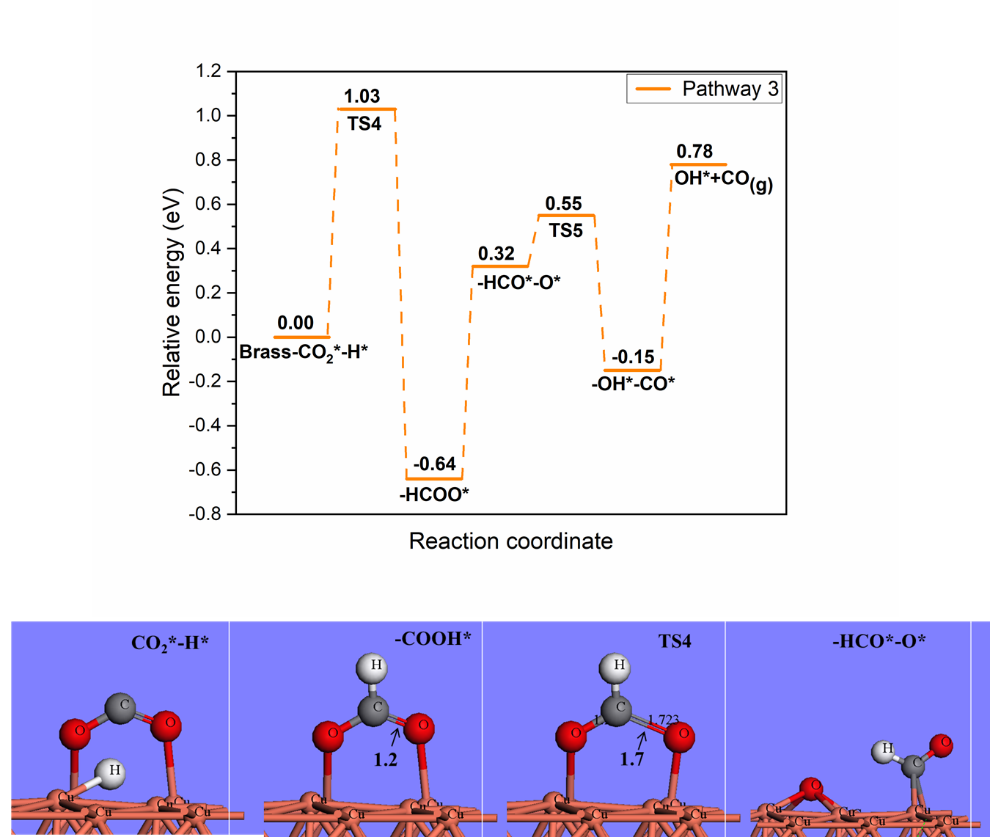


**Figure S13.** Potential energy profile for the **H-assisted dissociation** of CO_2_ via the **HCOO*** path on the CuZn30% (111) surface.

**References**

1. Connor, Paula, Jona Schuch, Bernhard Kaiser, and Wolfram Jaegermann. 2020. “The Determination of Electrochemical Active Surface Area and Specific Capacity Revisited for the System MnOx as an Oxygen Evolution Catalyst.” *Zeitschrift Für Physikalische Chemie* 234(5):979–94. doi: doi:10.1515/zpch-2019-1514.
2. Nunomura, Norio. n.d. “Density Functional Theory Study of Cu-Zn Alloys.” 902012035:7–10.
3. Varandili, Seyedeh Behnaz, Dragos Stoian, Jan Vavra, Kevin Rossi, James R. Pankhurst, Yannick T. Guntern, Núria López, and Raffaella Buonsanti. 2021. “ Elucidating the Structure-Dependent Selectivity of CuZn towards Methane and Ethanol in CO_2_ Electroreduction Using Tailored Cu/ZnO Precatalysts .” *Chemical Science* 12(43):14484–93. doi: 10.1039/d1sc04271h.
